# The Neighborhood Food Environment and the Onset of Child-Hood Obesity: A Retrospective Time-Trend Study in a Mid-sized City in China

**DOI:** 10.3389/fpubh.2021.688767

**Published:** 2021-07-26

**Authors:** Peiling Zhou, Ruifang Li, Kun Liu

**Affiliations:** ^1^School of Architecture, Harbin Institute of Technology (Shenzhen), Shenzhen, China; ^2^Shenzhen Key Laboratory of Urban Planning and Decision Making, Harbin Institute of Technology (Shenzhen), Shenzhen, China

**Keywords:** childhood obesity, built environment, neighborhood, food environment, China

## Abstract

Nowadays, obesity and its associated chronic diseases have become a steadily growing public health problem, spreading from the older to younger age groups. Studies have contended that the built environment, particularly the food environment and walkability, may contribute to the prevalence of childhood obesity. In Asian countries which are characterized by rapid urbanization, high population density and oriental diets, little is known about how such urban built environment affects the onset of childhood obesity. This study juxtaposes the effect of food environment, walkability, and outdoor activity spaces at the neighborhood level upon childhood body weight in a mid-sized city in China. This observational study utilizes a retrospective time-trend study design to examine the associations between neighborhood built environment and children's body weight in Zhanjiang City, a mid-sized city in Guangdong Province, China. Robust multiple linear and logistic regression models were used to estimate associations between the built environments and child BMI and weight status (i.e., overweight/obesity and obesity only). This study finds that: (1) Western-style fast food and Chinese-style fast food have divergent impacts on childhood body weight. At neighborhood level, while increased exposure to Western-style fast food may increase child BMI and the risk of overweight and obesity, increased exposure to Chinese-style fast food, on the contrary, may reduce child BMI and the risk of overweight and obesity, indicating a positive health impact of Chinese-style fast food. (2) However, the positive health impacts brought about by Chinese-style fast food, walkable environments and accessible traditional fruit/vegetable markets have gradually disappeared in recent years. This study is among the first to simultaneously consider the divergent and changing impact of food environment upon childhood body weight in urban China. The findings provide important implications for healthy city design and the management of food retail industry in addressing the obesity epidemic in younger generations living in Asian cities. As prominent differences exist in food culture between Asian and Western cities, more attention should be paid to healthy food environment in future studies and related urban planning strategies formulation.

## Introduction

Obesity has been proved to be a prominent risk factor for metabolic syndromes, which may develop chronic diseases such as hypertension, diabetes, coronary heart disease and stroke (1). In recent years, obesity and its associated chronic diseases have become a steadily growing public health problem, spreading from the older to younger age groups worldwide. In China, the rates of childhood overweight have increased to 14.0% in boys and 9.5% in girls (2). Obese children are about five times more susceptible to adult obesity than non-obese children (3). Childhood obesity has become a potential health risk of Chinese urban residents and has aroused great concern.

While the causes of childhood overweight and obesity are complex, including genetics (4), environmental (5) and energy-balance dysregulation (6–8), one of the noticeable risks of current obesity epidemic is the urban environment characteristics cause overwhelmed calorie intake, reducing physical activity which causes reducing metabolic rate and reducing energy expenditure (9). Lifestyle and behavioral interventions are proved to be ineffective for weight-loss (8, 10). Children living in densely developed urban areas, where they have easy access to energy packed food and little need for energy expenditure activities, are commonly exposed to the risk which is hard to be solved (6, 10). It is therefore important to study the association between urban built environment and childhood obesity.

The impacts of built environment on body size have been evaluated from different perspectives. General land use indices, such as urban sprawl and land use mix are found consistently associated with obesity prevalence (11, 12). At neighborhood level, studies have paid more attention to how the density, walkability and accessibility of physical activity facilities affect people's energy expenditure and body size by shaping their physical activity behaviors (13, 14). A group of studies has focused on neighborhood food environment and body size, in view of the vital role that food environment plays in shaping people's eating behaviors and energy intakes (15). While studies have identified main neighborhood-level food environment that may affect the prevalence of obesity, including the availability of groceries, supermarkets (16, 17), fast food (18–20), and food outlets (21, 22), findings yet remain to be inconsistent, with variation by country, scale and age group.

In particular, findings in the studies of children are somewhat inconsistent with that of adults. For example, while studies have found that mixed land use, walkability, accessible destinations, and proper active commuting to school may increase children's physical activity, which in turn reduce the prevalence of childhood obesity (23–25), there is no evidence of association between the availability of supermarkets, fast food and food outlets and childhood obesity, except the inevitable positive association between fast food availability and obesity among children from low-income households (22). Comparatively, the positive association between the availability of convenience stores and obesity and the negative association between the availability of fruit/vegetable markets and obesity have been observed among children (26). Children's body size is also affected by the social environment of neighborhood. For example, low neighborhood socioeconomic status (SES) may lead to a less diverse food environment and hence increase the risk of childhood obesity (27), while high neighborhood SES are associated with better physical activity environment and low childhood obesity morbidity (28). Apart from the neighborhood environment, school environment and policies are found to be important in affecting child body size (29, 30).

However, previous studies that examine the effect of fast food availability on childhood obesity usually consider fast food availability as an index of unhealthy food environment without taking into account the diversity nor the development of fast food in the Asian society (31). It is partially because of the fact that most of these studies were conducted in Western countries, where fast food restaurants are relatively homogeneous—serving food of excessive size and high in fat, salt and sugar plus low in fiber and vegetable (such as burgers and French fries) (32, 33). Fast food restaurants in other parts of the world, like China, serve food of greater diversity, in terms of food choices, cooking methods and nutrition structures. Compared with Western-style fast food offered at fast food chains such as KFC, McDonald, and Burger King, Chinese-style fast food, prepared with traditional Chinese cooking methods, consists of a larger proportion of steamed item and vegetables (34). Despite significant loss of vitamins due to Chinese cooking methods and relatively low protein supply, Chinese fast food restaurants provide a more comprehensive nutrition environment and accessibility to balanced nutrition intake (34, 35). Fast food consumption is gaining popularity and registers a faster growth among children and adolescents than in other age groups in China (36), and thus its health impact on children needs further estimation.

The development of Chinese fast food is in parallel with the rapid urbanization of China over the past two decades (36). While Chinese-style fast food has once been proved to be healthy, it is seeing a gradual change in cooking methods in face of competition from Western-style fast food (36, 37). Cheap capitalism, which is characterized by low price, inferior food quality and degraded business ethics (37), has gradually dominated Chinese fast food industry, leading to increased health risks. Current fast-paced lifestyle also leads to frequent consumption of fast food at restaurants and instant food at convenient stores instead of cooking at home (36). Groceries markets in China are also different from those of Western countries. Available in larger scale and higher density, groceries markets, existing in China in form of fruit/vegetable markets, have long been the major food sourcing outlets for households in China. However, urban sprawl and urban renewal have driven many fruit/vegetable markets away from urban residential neighborhoods. Change in the food supply chain also leads to changed quality of food in fruit/vegetable market (38). Moreover, rapid urbanization and high population density in China also have unexpected impact on physical activity environment. With rapid urbanization, high population density and typical domestic diet in China, little is known about whether and which aspect of China's urban built environment accounts for the onset of childhood obesity.

Based on a retrospective time-trend study on a mid-sized city in China, this study aims to examine the associations between multiple neighborhood built environments, particularly food environment, and children's body size. Given the significant characteristics and rapid development of Chinese fast food in recent years, simultaneously investigating both Chinese and Western types of fast food may yield a more precise understanding of its health impact. The findings of this study provide important implications for healthy cities design and the management of food retail industry in addressing the obesity prevalence in younger generations in urban societies.

## Methods

### Study Design

**Figure 1:**
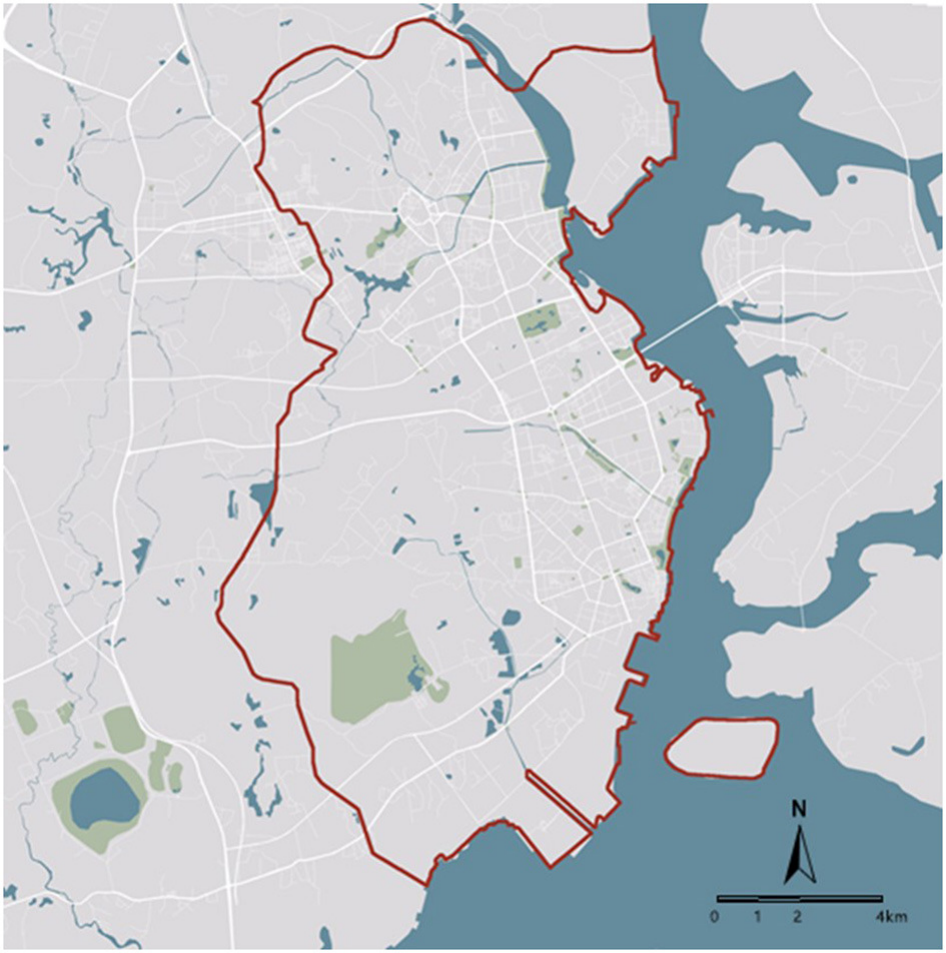
Research sites: Chikan District and Danxia District, two urban areas in Zhanjiang.

This observational study utilizes a retrospective time-trend study design to examine the associations between neighborhood built environment and children's body size. This study took place in Zhanjiang City, a mid-sized city in Guangdong Province, China. Zhanjiang had a population of 8.48 million in 2019 (39). With the support from municipal administration, the fast food industry in Zhanjiang has rapidly developed since 2010 (40). In 2016, the total revenue of food catering industry increased to 2.19 billion US dollars, with 38.7% from fast food restaurants (41). The rapid development of fast food industry came along with sanitary and health problems, for which a new standard governing fast food catering was announced by the municipal Food and Drug Administration (FDA) (42). The researchers were given permission to analyze the three-waves health survey data from 2011 to 2016 of residents in two areas in Zhanjiang City, namely Chikan District and Danxia District (Figure 1), including children's health survey data. There were *N* = 8,488 children observations aged 6–15 in this dataset.

This study addresses the following questions:

1. What were the associations between childhood body size and neighborhood built environment?
2. Do the associations between childhood body size and neighborhood built environment vary between children in primary schools and middle schools?

### Data Sources and Population

The health survey data was collected by community health service centers, which are the primary urban healthcare units in China. From 2011 to 2016, the community health service center conducted three waves' health surveys, the first wave from 2011–2012, the second from 2013 to 2014, and the third from 2015 to 2016. Although the community health service centers conduct health surveys every other year for residents, compiled cross-year individual identification code is not provided for longitudinal data tracking. Therefore, the health survey data is comprised of three retrospective cross-sectional cohorts; admitted to the health survey taken from 2011 to 2012 (1st round), from 2013 to 2014 (2nd round) and from 2015 to 2016 (3rd round). There are no repeated measurements for each child. Since this research aims to study the associations between neighborhood built environment and childhood overweight, only samples with identified residential addresses and height/weight data were considered. The final dataset comprised 7,350 observations aged 6–15 for this study.

The built environment data include land use data, street map, and POI data. Considering that the observations span from 2011 to 2016, it would be ideal to use built environment data across the years. However, with the limitations on data accessibility, we only use land use data, street map, and POI data in 2016. All the built environment data were imported into ArcGIS 10.4 (43) and joint with observations by their residential address.

### Measures

#### Outcome Variables

The body mass index, BMI (in kg/m^2^) was calculated by body size and height measured by nurses using standardized measurement devices. Since children's body size varies by age, sex, ethnicity and other factors, the definition of childhood overweight and obesity varies across the world. For example, in the U.S., childhood overweight and obesity was defined as sex-age-specific BMI > 85th and 95th percentile of the 2000 CDC Growth Chart, respectively (44). Given that the body mass growth of Chinese children is different from that of other countries (45), childhood overweight and obesity was defined as sex-age-specific BMI > 85th and 95th percentile of the WS/T586-2018, the Chinese National Standard of Overweight and obesity screening for school-age children and adolescents (46, 47), respectively.

#### Built Environment Variables

The built environment variables comprised food environment factors and physical activity environment factors. Food environment factors included the density of five different categories of food outlets: fruit/vegetable market density, supermarket density, convenient store density, Chinese-style and Western-style fast food restaurant densities. These food outlets were extracted from POI datasets and geocoded in the land use map of Zhanjiang. Physical activity environment factors included land use mix and the density of four different categories of land use: main roads, side roads, residences and public parks. To consider the transport-related physical activity of children, the distance to school was also deemed as a factor of physical activity environment. Only the food outlets and land use within 500 meters from each observation's residential address were considered in our density calculation.

#### Covariates

Individual-level covariates included age and gender. Neighborhood-level covariates included socioeconomic status (SES) and urbanicity of residence. The urbanicity of residence in Zhanjiang City consists of two types: urban and urban village. The urban village represents the under-urbanized neighborhoods within urban area. As parental education level and household income data is not available in the health survey dataset, we use the continuous measure of house prices per area unit of the observations' neighborhood to represent neighborhood-level SES.

### Data Analysis

Chi-square tests and *t*-tests were conducted to significant disparities in children's sociodemographic, body size and built environment characteristics between children in primary schools and in middle schools. Linear and logistic mixed model were used to estimate associations between the built environments and child BMI and weight status (i.e., overweight/obesity and obesity only) across the year, including a random effect for the year. Robust multiple linear and logistic regression models were used to estimate associations between the built environments and child BMI and weight status (i.e., overweight/obesity and obesity only) in each wave. Model 1–12 estimated the associations between neighborhood built environments and child BMI (**Table 2**); Model 13–24 were for childhood overweight and obesity (**Table 3**); Model 25–36 were for childhood obesity only (**Table 3**). Model 1–4 were for all the children aged 6–15; model 5–8 were for primary school children; Model 9–12 were for middle school children. With the rapid growth of Western-style fast food restaurants since 2010, the associations were estimated every 2 years (per wave) in other models (i.e., Model 2, 6, and 10 for wave 1 taken from 2011 to 2012, Model 3, 7, and 11 for wave 2 taken from 2013 to 2014, Model 4, 8, 12 for wave 3 taken from 2015 to 2016). All the models have been adjusted for children's age, gender, urbanicity, and neighborhood SES. To adjust for multiple comparisons, the raw *P*-value was adjusted using the FDR method (48). All the statistical analyses were conducted using Stata 14 (49).

## Results

### Descriptive Analyses

Table 1 showed the sociodemographic characteristics, weight status and built environment characteristics of the observed children. The average age of the observations was 11.5 years old. There exists a significant difference in weight status between primary school and middle school children. The prevalence of both overweight and obesity were significantly higher among primary school children (41.5 vs. 9.6, 17.5 vs. 0.6, respectively). From 2011 to 2016, the prevalence of both overweight and obesity significantly increased (19.4 to 30.4, 6.9 to 13.4, respectively). Compared with primary school children, middle school children observations lived in neighborhoods with relatively lower SES and longer distance to school. The physical activity environments and food environments were similar between neighborhoods of primary and middle school children.

**Table 1:** Sociodemographic characteristics, weight status, and built environment of the children.

	**% or Mean**			
**Variables**	**All^**a**^**	**Primary School**	**Middle School**	**p-value^**b**^**
Gender				0.673
%Boys	56.4	56.6	56.1	
%Girls	43.6	43.4	43.9	
Age (average)	11.5	9.5	14.0	0.000
BMI (average)	19.2	18.8	19.7	0.000
Weight status				
%Overweight/obesity	25.8	38.4	9.8	0.000
%Obesity	10.9	18.9	0.7	0.000
Urbanicity				0.001
%Urban	87.1	88.2	85.6	
%Urban village	12.9	11.8	14.4	
Neighborhood SES (average)	6,471	6,568	6,348	0.000
Food environment				
Fruit/vegetable market (average)	1.5	1.4	1.5	0.000
Supermarket (average)	1.7	1.7	1.7	0.741
Convenient store (average)	2.3	2.3	2.3	0.979
Chinese fast food (average)	1.9	1.9	1.9	0.577
Western fast food (average)	1.6	1.6	1.5	0.998
Physical activity environment				
Land use mix (average)	0.5	0.5	0.5	0.998
Main road density (average)	1.5	1.6	1.5	1.000
Side road density (average)	2.9	2.9	2.9	0.016
Residence density (average)	0.5	0.5	0.5	0.089
Public park density (average)	0.7	0.8	0.7	1.000
Distance to school (average)	606	404	863	0.000
Total number *N*	8,441	4,717	3,724	

a *The percentage of each category for the categorical variables was count and the average values of the continuous variables were calculated*.

b *P-values tested the differences in each variable between primary school children and middle school children and were based on Chi-square tests for categorical variables or t-tests for continuous variables*.

Linear regressions showed an association between the food environment SES. Neighborhoods with higher SES are associated with fewer Chinese fast food (β = −0.15, 95%CI:−0.16, −0.13, *p* < 0.001), Western fast food (β = −0.05, 95%CI:−0.06, −0.04, *p* < 0.001), fruit/vegetable markets (β = −0.16, 95%CI:−0.18, −0.15, *p* < 0.001), and supermarkets (β = −0.11, 95%CI:−0.12, −0.09, *p* < 0.001).

### Associations of Neighborhood Environment and Childhood Weight Status

The left parts of Tables 2–**4** showed the associations of neighborhood built environments and child BMI, childhood overweight/obesity and childhood obesity, respectively. (1) After FDR adjustment, children living in neighborhoods with higher SES tended to have lower BMI (Table 2), while the significant association was not observed for overweight and obesity risk. (2) No significant associations of urbanity and childhood weight status were observed.

**Table 2:** Associations of neighborhood built environments and child body mass index (BMI) 2011-2016^a^.

**Built Environment**	**All (*****N*** **=** **8,441)**				**Primary School (*****N*** **=** **4,717)**				**Middle School (*****N*** **=** **3,724)**			
	**Overall^**b**^**	**Wave 1**	**Wave 2**	**Wave 3**	**Overall**	**Wave 1**	**Wave 2**	**Wave 3**	**Overall**	**Wave 1**	**Wave 2**	**Wave 3**
Urbanicity^c^	−0.02	0.04	0.43	−0.71^*d^	0.11	0.59	0.36	−0.90^*^	−0.15	−0.36^*^	0.34^**^	0.06
	−0.25,0.21	−0.29,0.36	−0.10,0.95	−1.26,−0.15	−0.23,0.45	0.06,1.13	−0.39,1.10	−1.62,−0.19	−0.44,0.13	−0.70,−0.02	−0.57,1.25	−0.75,0.87
	0.882	0.819	0.111	0.013	0.523	0.031	0.344	0.013	0.293	0.013	0.008	0.735
	0.738	0.701	0.189	0.037	0.541	0.071	0.417	0.037	0.372	0.037	0.025	0.655
Neighborhood SES	−0.16^***^	−0.11	−0.24^***^	−0.28^*^	−0.15^**^	−0.04	−0.19	−0.36^*^	−0.17^***^	−0.14	−0.33	−0.06
	−0.23,−0.09	−0.21,– 0.00	−0.38,−0.10	−0.51,−0.53	−0.26,−0.05	−0.20,0.13	−0.37,−0.01	−0.65,−0.07	−0.27,−0.08	−0.25,−0.03	−0.57,−0.09	−0.40,0.28
	0.000	0.041	0.001	0.016	0.005	0.666	0.035	0.015	0.000	0.038	0.464	0.880
	0.001	0.089	0.004	0.043	0.017	0.621	0.078	0.041	0.001	0.083	0.506	0.738
**Food environment**												
Fruit/vegetable market	−0.17^***^	−0.24^***^	−0.20	0.03	−0.27^***^	−0.38^***^	−0.36^*^	0.07	−0.05	−0.05	−0.03	−0.12
	−0.27,−0.08	−0.36,−0.12	−0.41,0.02	−0.36,0.43	−0.41,−0.13	−0.57,−0.18	−0.67,−0.06	−0.47,0.62	−0.17,0.07	−0.17,0.07	−0.38,0.31	−0.57,0.32
	0.000	0.000	0.075	0.870	0.000	0.000	0.019	0.788	0.410	0.443	0.848	0.585
	0.001	0.001	0.143	0.732	0.001	0.001	0.049	0.697	0.472	0.499	0.721	0.598
Supermarket	0.04	0.35^***^	−0.31^***^	−0.21	0.20	0.57^***^	−0.32	−0.06	−0.05	0.30^**^	−0.20	−0.49
	−0.08,0.15	0.18,0.53	−0.48,−0.13	−0.70,0.28	0.02,0.38	0.30,0.83	−0.61,−0.03	−0.67,0.56	−0.19,0.10	0.09,0.51	−0.49,0.08	−1.28,0.30
	0.55	0.000	0.001	0.392	0.030	0.000	0.030	0.853	0.546	0.006	0.162	0.222
	0.56	0.001	0.004	0.453	0.070	0.001	0.070	0.725	0.557	0.019	0.255	0.312
Convenient store	0.10	−0.06	0.23^*^	0.13	0.17	0.04	0.30^*^	0.15	0.04	−0.07	0.13	0.16
	−0.03,0.22	−0.22,0.10	0.05,0.41	−0.40,0.66	−0.01,0.37	−0.23,0.31	0.05,0.55	−0.51,0.80	−0.11,0.20	−0.25,0.11	−0.17,0.44	−0.72,1.03
	0.137	0.452	0.014	0.626	0.068	0.795	0.018	0.663	0.586	0.452	0.395	0.723
	0.224	0.501	0.039	0.608	0.134	0.699	0.047	0.621	0.598	0.501	0.454	0.654
Chinese fast food	−0.41^***^	−0.53^***^	−0.53^***^	0.05	−0.62^***^	−0.88^***^	−0.66^**^	0.03	−0.15	−0.16	−0.34	0.19
	−0.55,−0.27	−0.72,−0.34	−0.82,−0.24	−0.50,0.60	−0.84,−0.40	−1.20,−0.56	−1.07,−0.24	−0.74,0.79	−0.32,0.03	−0.35,0.03	−0.71,0.03	−0.56,0.94
	0.000	0.000	0.000	0.87	0.000	0.000	0.002	0.947	0.101	0.105	0.075	0.618
	0.001	0.001	0.001	0.732	0.001	0.001	0.008	0.775	0.177	0.183	0.143	0.607
Western fast food	0.30^**^	0.03	0.23	1.07^**^	0.39^**^	−0.01	0.34	1.15^*^	0.18	0.08	0.06	0.85
	0.11,0.49	−0.28,0.33	−0.14,0.60	0.36,1.78	0.11,0.68	−0.54,0.51	−0.05,0.73	0.19,2.12	−0.05,0.42	−0.24,0.39	−0.66,0.79	−0.13,1.83
	0.002	0.866	0.223	0.003	0.006	0.962	0.09	0.019	0.132	0.633	0.868	0.088
	0.008	0.732	0.312	0.011	0.019	0.776	0.16	0.049	0.219	0.609	0.732	0.157
**Physical activity environment**												
Land use mix	−0.29	−1.58^*^	0.41	−0.17	−0.70	−2.42	0.05	−0.60	0.02	−0.76	0.50	−0.97
	−1.22,0.64	−2.90,−0.26	−1.29,2.11	−3.92,3.58	−2.13,0.71	−4.53,−0.31	−2.03,2.13	−5.70,4.50	−1.10,1.14	−2.05,0.53	−2.40,3.39	−6.22,4.27
	0.455	0.019	0.636	0.929	0.329	0.024	0.962	0.817	0.979	0.249	0.737	0.715
	0.501	0.049	0.609	0.763	0.405	0.058	0.776	0.701	0.782	0.334	0.655	0.649
Main road density	0.04	0.20^*^	−0.04	−0.37	0.19	0.43^**^	0.01	−0.42	−0.11	−0.02	−0.10	−0.28
	−0.07,0.14	0.04,0.37	−0.23,0.15	−0.74,0.00	0.03,0.35	0.16,0.69	−0.25,0.26	−0.87,0.04	−0.25,0.02	−0.20,0.16	−0.40,0.20	−0.87,0.31
	0.521	0.012	0.664	0.047	0.024	0.002	0.963	0.071	0.085	0.837	0.518	0.351
	0.541	0.035	0.621	0.098	0.058	0.008	0.776	0.137	0.156	0.710	0.541	0.418
Side road density	−0.17^***^	−0.20^***^	−0.14^**^	0.13	−0.26^***^	−0.38^***^	−0.13	0.15	−0.06	−0.03	−0.16^*^	0.15
	−0.24,−0.11	−0.31,−0.10	−0.25,−0.03	−0.12,0.38	−0.36,−0.16	−0.55,−0.21	−0.31,0.05	−0.18,0.47	−0.14,0.03	−0.15,0.10	−0.29,−0.03	−0.21,0.51
	0.000	0.000	0.010	0.308	0.000	0.000	0.167	0.381	0.193	0.645	0.015	0.419
	0.001	0.001	0.030	0.384	0.001	0.001	0.26	0.442	0.285	0.615	0.041	0.472
Residence density	0.30	1.20	−0.59	−2.82	0.00	1.24	−0.50	−3.34	0.74	1.31	−0.49	−3.08
	−0.57,1.11	0.08,2.33	−2,18,1.01	−6.10,0.47	−1.27,1.28	−0.63,3.11	−2.51,1.50	−7.70,1.01	−0.26,1.76	0.20,2.41	−3.57,2.60	−8.45,2.29
	0.530	0.036	0.473	0.093	0.995	0.194	0.622	0.132	0.150	0.021	0.757	0.26
	0.541	0.080	0.512	0.165	0.783	0.285	0.607	0.219	0.239	0.053	0.67	0.341
Public park density	−0.52^***^	−0.21	−0.48^***^	−0.94^***^	−0.57^***^	−0.05	−0.54^***^	−1.30^***^	−0.42^***^	−0.37^***^	−0.33^**^	0.02
	−0.64,−0.40	−0.39,−0.03	−0.67,−0.30	−1.33,−0.54	−0.75,−0.39	−0.33,0.24	−0.82,−0.27	−1.79,−0.80	−0.57,−0.27	−0.58,−0.17	−0.56,−0.10	−0.58,0.62
	0.000	0.024	0.000	0.000	0.000	0.756	0.000	0.000	0.000	0.000	0.005	0.949
	0.001	0.058	0.001	0.001	0.001	0.670	0.001	0.001	0.001	0.001	0.017	0.775
Distance to school	0.02	0.21^***^	−0.03	−0.20	0.25	0.60^***^	0.18	0.00	0.04	0.27^***^	−0.07	−0.24
	−0.08,0.13	0.08,0.34	−0.26,0.20	−0.67,0.27	0.03,0.47	0.28,0.92	−0.17,0.53	−0.94,0.93	−0.07,0.15	0.12,0.41	−0.44,0.30	−0.79,0.31
	0.650	0.001	0.799	0.397	0.027	0.000	0.305	0.992	0.532	0.000	0.694	0.388
	0.618	0.004	0.699	0.454	0.064	0.001	0.383	0.783	0.541	0.001	0.641	0.448
Constant	19.20	20.09	19.67	18.26	19.27	20.22	19.83	19.11	16.61	15.99	17.52	17.23

a *All models were adjusted for age, gender, neighborhood socioeconomic status, and urbanicity*.

b *The overall model reports the result of a linear mixed regression model including a random effect for the three waves. The Wave 1–3 models reports the results of linear regression models for wave 1–3, respectively. Wave 1 represent the cohort that took the health survey from 2011 to 2012; Wave 2 represent the cohort that took the health survey from 2013 to 2014; Wave 3 represent the cohort that took the health survey from 2015 to 2016*.

c *For each variable, the first row reports the coefficient; the second row reports the 95% confidence interval, the third row reports the naïve p-value; the fourth row reports the FDR sharpened q-value*.

d *p < 0.05 and q < 0.02, **p < 0.01 and q < 0.03, ***p < 0.001 and q < 0.004.

### Food Environment

After FDR adjustment, children living in neighborhoods with high density of fruit/vegetable market showed a lower BMI (Table 2) and less likely to be overweight (Table 3), while the association was not observed for obesity risk. The associations of high density of fruit/vegetable market and low BMI and overweight risk were significantly observed in 2011–2012 (Tables 2, 3) but disappeared since 2013. After FDR adjustment, the associations of supermarket density and childhood weight status were inconsistent between years. In 2011–2012, children living in neighborhoods with high supermarket density showed a higher BMI (Table 2) and were more likely to be overweight (Table 3) and obese (Table 4), while in 2013–2014, children living in neighborhoods with high supermarket density showed a lower BMI (Table 2), and were less likely to be overweight (Table 3). No significant associations of supermarket density and childhood weight status were observed in 2015–2016. After FDR adjustment, no significant associations were observed between convenient store density and childhood body size.

**Table 3:** Associations of neighborhood built environments and childhood overweight and obesity 2011–2016^a^.

**Built Environment**	**All (*****N*** **=** **8,441)**				**Primary School (*****N*** **=** **4,717)**				**Middle School (*****N*** **=** **3,724)**			
	**Overall^**b**^**	**Wave 1**	**Wave 2**	**Wave 3**	**Overall**	**Wave 1**	**Wave 2**	**Wave 3**	**Overall**	**Wave 1**	**Wave 2**	**Wave 3**
Urbanicity^c^	1.03	1.17	1.66	0.46 ^*d^	1.12	1.75	1.55	0.46^**^	0.84	0.59	1.31^*^	0.90
	0.83,1.26	0.83,1.65	1.09,2.54	0.29,0.74	0.88,1.42	1.13,2.70	0.91,2.63	0.28,0.78	0.54,1.28	0.32,1.06	0.58,2.94	0.17,4.85
	0.792	0.414	0.033	0.015	0.366	0.919	0.227	0.01	0.415	0.195	0.018	0.96
	0.699	0.472	0.075	0.041	0.429	0.755	0.314	0.03	0.472	0.285	0.047	0.776
Neighborhood SES	0.92	0.96	0.86^*^	0.78^***^	0.94	1.01^*^	0.90	0.75^**^	0.86	0.88	0.73	0.98
	0.86,0.99	0.88,1.06	0.75,0.99	0.64,0.95	0.87,1.01	0.9,1.13	0.76,1.07	0.60,0.93	0.74,1	0.73,1.07	0.57,0.95	0.54,1.81
	0.022	0.364	0.018	0.001	0.109	0.012	0.107	0.004	0.048	0.077	0.519	0.901
	0.055	0.428	0.047	0.004	0.188	0.035	0.185	0.014	0.1	0.145	0.541	0.739
**Food environment**												
Fruit/vegetable market	0.84^***^	0.76^***^	0.80	1.14	0.81^***^	0.70^***^	0.72^*^	1.19	0.89	0.93	1.00	0.91
	0.77,0.92	0.68,0.84	0.65,0.99	0.82,1.58	0.73,0.89	0.62,0.80	0.57,0.93	0.83,1.69	0.75,1.06	0.74,1.15	0.65,1.55	0.36,2.33
	0.000	0.000	0.041	0.428	0.000	0.000	0.011	0.341	0.205	0.486	0.983	0.846
	0.001	0.001	0.089	0.485	0.001	0.001	0.033	0.417	0.294	0.518	0.783	0.721
Supermarket	1.05	1.51^***^	0.65^***^	0.83	1.11	1.56^***^	0.65^***^	0.93	1.00	1.64^**^	0.78	0.38
	0.94,1.18	1.28,1.78	0.53,0.80	0.59,1.17	0.98,1.27	1.28,1.91	0.50,0.85	0.65,1.34	0.80,1.26	1.18,2.29	0.54,1.15	0.14,1.00
	0.385	0.000	0.000	0.287	0.105	0.000	0.001	0.707	0.977	0.004	0.208	0.049
	0.445	0.001	0.001	0.365	0.183	0.001	0.004	0.645	0.782	0.014	0.297	0.101
Convenient store	1.06	0.89	1.20	1.03	1.09	0.90	1.29	1.10	1.11	1.02	1.03	0.52
	0.94,1.19	0.76,1.05	0.96,1.49	0.66,1.62	0.95,1.25	0.74,1.11	1.00,1.66	0.67,1.82	0.88,1.40	0.77,1.36	0.64,1.66	0.12,2.22
	0.370	0.181	0.111	0.890	0.234	0.334	0.047	0.699	0.358	0.874	0.918	0.375
	0.432	0.273	0.189	0.738	0.324	0.409	0.098	0.642	0.423	0.734	0.755	0.434
Chinese fast food	0.68^***^	0.57^***^	0.55^***^	0.89	0.68^***^	0.54^***^	0.58^**^	1.01	0.67^**^	0.70	0.55	0.58
	0.59,0.78	0.47,0.70	0.40,0.76	0.58,1.36	0.58,0.80	0.42,0.69	0.39,0.85	0.62,1.66	0.51,0.88	0.51,0.97	0.30,0.99	0.18,1.85
	0.000	0.000	0.000	0.586	0.000	0.000	0.005	0.956	0.005	0.033	0.047	0.356
	0.001	0.001	0.001	0.598	0.001	0.001	0.017	0.776	0.017	0.075	0.098	0.421
Western fast food	1.34^***^	1.01	1.25	2.70^***^	1.34^**^	0.96	1.29	2.63^***^	1.37	1.10	1.16	2.91
	1.12,1.60	0.76,1.34	0.93,1.67	1.58,4.62	1.10,1.64	0.67,1.37	0.92,1.82	1.48,4.66	0.96,1.97	0.65,1.86	0.67,2.02	0.83,10.18
	0.001	0.949	0.141	0.000	0.004	0.818	0.137	0.001	0.086	0.727	0.602	0.095
	0.004	0.775	0.228	0.001	0.014	0.701	0.224	0.004	0.156	0.655	0.603	0.167
**Physical activity environment**												
Land use mix	0.59	0.14^***^	0.65	1.28	0.53	0.07^***^	0.69	1.17	0.58	0.29	0.41	0.11
	0.25,1.37	0.05,0.42	0.10,4.16	0.07,23.29	0.19,1.47	0.02,0.30	0.08,6.00	0.04,38.01	0.11,3.01	0.03,2.69	0.01,13.43	0.00,1438.97
	0.222	0.000	0.653	0.868	0.222	0.000	0.735	0.930	0.517	0.276	0.619	0.645
	0.312	0.001	0.620	0.732	0.312	0.001	0.655	0.763	0.541	0.355	0.607	0.615
Main road density	1.06	1.26^**^	1.07	0.73	1.10	1.31^*^	1.06	0.70	1.01	1.15	1.07	1.20
	0.96,1.17	1.07,1.48	0.89,1.29	0.55,0.98	0.98,1.24	1.06,1.61	0.85,1.32	0.51,0.97	0.82,1.24	0.86,1.55	0.76,1.52	0.53,2.75
	0.226	0.006	0.454	0.034	0.100	0.011	0.614	0.034	0.928	0.347	0.699	0.658
	0.313	0.019	0.501	0.077	0.175	0.033	0.607	0.077	0.763	0.417	0.642	0.621
Side road density	0.86^***^	0.84^***^	0.87^*^	1.18	0.85^***^	0.79^***^	0.95	1.17	0.89	0.93	0.70^***^	1.36
	0.81,0.92	0.75,0.93	0.78,0.98	0.99,1.41	0.79,0.92	0.69,0.90	0.82,1.09	0.96,1.43	0.79,1.01	0.78,1.11	0.58,0.85	0.92,2.01
	0.000	0.001	0.020	0.060	0.000	0.000	0.457	0.118	0.082	0.413	0.000	0.127
	0.001	0.004	0.050	0.119	0.001	0.001	0.502	0.199	0.152	0.472	0.001	0.212
Residence density	0.67	1.46	0.52	0.13	0.60	1.12	0.71	0.11	0.90	2.06	0.42	0.02
	0.31,1.43	0.55,3.89	0.10,2.76	0.01,1.62	0.24,1.50	0.30,4.22	0.10,5.18	0.01,2.13	0.21,3.88	0.30,14.12	0.02,8.56	0.00,16.22
	0.303	0.449	0.443	0.112	0.277	0.871	0.736	0.143	0.886	0.462	0.575	0.249
	0.382	0.501	0.499	0.190	0.356	0.732	0.655	0.230	0.738	0.506	0.591	0.334
Public park density	0.62^***^	0.93	0.63^***^	0.48^***^	0.59^***^	0.98	0.62^***^	0.41^***^	0.72^**^	0.86	0.71	1.29
	0.55,0.69	0.78,1.10	0.50,0.79	0.34,0.69	0.52,0.67	0.79,1.22	0.48,0.80	0.28,0.60	0.57,0.92	0.63,1.18	0.44,1.12	0.32,5.17
	0.000	0.382	0.000	0.000	0.000	0.886	0.000	0.000	0.009	0.354	0.142	0.718
	0.001	0.442	0.001	0.001	0.001	0.738	0.001	0.001	0.027	0.42	0.229	0.65
Distance to school	0.86^**^	1.00	0.86	0.69	1.03	1.16	1.17	1.00	0.76^***^	1.06	0.72	0.31^**^
	0.77,0.95	0.86,1.16	0.71,1.04	0.46,1.03	0.88,1.20	0.92,1.47	0.84,1.62	0.57,1.75	0.65,0.90	0.84,1.34	0.52,0.99	0.14,0.69
	0.004	0.991	0.127	0.070	0.719	0.211	0.348	1.000	0.001	0.633	0.044	0.004
	0.014	0.783	0.212	0.137	0.650	0.300	0.417	0.785	0.004	0.609	0.093	0.014
Constant	187.38	332.25	758.64	63.16	141.97	454.83	350.40	39.10	342.07	136.99	980.30	1.38e+7

a *All models were adjusted for age, gender, neighborhood socioeconomic status, and urbanicity*.

b *The overall model reports the result of a logistic mixed regression model including a random effect for the three waves. The Wave 1–3 models reports the results of logistic regression models for wave 1–3, respectively. Wave 1 represent the cohort that took the health survey from 2011 to 2012; Wave 2 represent the cohort that took the health survey from 2013 to 2014; Wave 3 represent the cohort that took the health survey from 2015 to 2016*.

c *For each variable, the first row reports the odds ratio; the second row reports the 95% confidence interval, the third row reports the naïve p-value; the fourth row reports the FDR sharpened q-value*.

d *p < 0.05 and q < 0.02, **p < 0.01 and q < 0.03, ***p < 0.001 and q < 0.004.

**Table 4:** Associations of neighborhood built environments and childhood obesity 2011–2016^a^.

**Built Environment**	**All (*****N*** **=** **8,441)**				**Primary School (*****N*** **=** **4,717)**				**Middle School (*****N*** **=** **3,724)**			
	**Overall^**b**^**	**Wave 1**	**Wave 2**	**Wave 3**	**Overall**	**Wave 1**	**Wave 2**	**Wave 3**	**Overall**	**Wave 1**	**Wave 2**	**Wave 3**
Urbanicity^c^	1.23	2.57	0.69	0.79	1.29	2.69	0.85	0.79	0.33	1.82	0.09	–
	0.90,1.68	1.37,4.83	0.35,1.35	0.45,1.40	0.94,1.78	1.41,5.13	0.42,1.72	0.44,1.45	0.09,1.21	0.09,35.49	0.00,2.79	
	0.196	0.668	0.059	0.106	0.119	0.523	0.083	0.120	0.095	0.137	0.110	
	0.286	0.621	0.118	0.184	0.200	0.541	0.154	0.201	0.167	0.224	0.189	
Neighborhood SES	0.89	1.03^**^	0.83	0.80	0.89	1.05^**^	0.84	0.80	0.75	0.71	0.07	–
	0.80,0.99	0.88,1.21	0.68,1.01	0.61,1.05	0.81,0.99	0.90,1.24	0.68,1.02	0.61,1.06	0.45,1.25	0.46,1.11	0.00,1.84	
	0.025	0.003	0.275	0.419	0.035	0.003	0.657	0.450	0.271	0.692	0.167	
	0.060	0.011	0.355	0.472	0.078	0.011	0.621	0.501	0.353	0.641	0.260	
**Food environment**												
Fruit/vegetable market	0.93	0.89	0.80	1.10	0.93	0.88	0.85	1.08	0.72	0.79	0.01^***^^c^	–
	0.82,1.06	0.74,1.07	0.59,1.08	0.71,1.71	0.82,1.06	0.73,1.07	0.63,1.15	0.68,1.71	0.38,1.37	0.43,1.45	0.00,0.13	
	0.279	0.199	0.140	0.664	0.320	0.194	0.294	0.752	0.319	0.449	0.001	
	0.358	0.289	0.228	0.621	0.396	0.285	0.372	0.667	0.396	0.501	0.004	
Supermarket	1.05	1.29	0.85	0.83	1.07	1.26	0.82	0.96	0.86	2.21	0.21	–
	0.89,1.25	1.00,1.68	0.63,1.14	0.50,1.37	0.90,1.27	0.96,1.66	0.60,1.11	0.59,1.56	0.36,2.04	0.58,8.41	0.00,25.63	
	0.542	0.052	0.272	0.467	0.452	0.090	0.205	0.861	0.727	0.246	0.526	
	0.552	0.105	0.353	0.507	0.501	0.160	0.294	0.732	0.655	0.333	0.541	
Convenient store	1.11	0.91	1.19	0.83	1.10	0.94	1.19	0.84	1.16	0.88	1.58	-
	0.92,1.32	0.70,1.18	0.88,1.60	0.49,1.43	0.92,1.33	0.72,1.23	0.88,1.61	0.48,1.46	0.59,2.51	0.31,2.50	0.00,698.56	
	0.260	0.479	0.265	0.510	0.303	0.664	0.269	0.531	0.749	0.810	0.883	
	0.341	0.518	0.345	0.536	0.382	0.621	0.351	0.541	0.665	0.700	0.738	
Chinese fast food	0.67^***^	0.47^***^	0.54^**^	1.11	0.69^***^	0.52^***^	0.51^**^	1.08	0.37	0.16^***^	0.23	–
	0.55,0.83	0.33,0.66	0.34,0.84	0.62,1.98	0.56,0.85	0.36,0.73	0.32,0.81	0.58,1.98	0.14,1.00	0.08,0.32	0.00,22.8	
	0.000	0.000	0.006	0.734	0.001	0.000	0.004	0.815	0.050	0.000	0.534	
	0.001	0.001	0.019	0.655	0.004	0.001	0.014	0.701	0.102	0.001	0.542	
Western fast food	1.68^***^	1.42	1.45	2.47^*^	1.60^***^	1.38	1.43	2.39	3.33	1.06	1.02	–
	1.29,2.14	0.87,2.30	0.97,2.18	1.21,5.05	1.24,2.08	0.83,2.28	0.95,2.16	1.12,5.08	1.04,10.61	0.13,8.56	0.01,75.74	
	0.000	0.161	0.073	0.013	0.000	0.210	0.087	0.024	0.042	0.957	0.993	
	0.001	0.254	0.140	0.037	0.001	0.299	0.157	0.058	0.090	0.776	0.783	
**Physical activity environment**												
Land use mix	2.43	0.94	2.04	5.36	2.14	0.88	1.41	4.02	125.25	0.33	1.29e+11	–
	0.69,8.50	0.15,6.06	0.18,22.93	0.11,261.19	0.60,7.69	0.14,5.35	0.12,16.15	0.07,235.89	0.16,100073.9	0.00,509.09	2.24e-08,7.47e+29	
	0.163	0.948	0.563	0.397	0.243	0.889	0.781	0.503	0.157	0.765	0.246	
	0.255	0.775	0.574	0.454	0.332	0.738	0.69	0.532	0.248	0.679	0.333	
Main road density	1.04	1.14	1.19	0.70	1.06	1.16	1.20	0.71	0.82	1.40	1.93	-
	0.90,1.21	0.86,1.51	0.93,1.54	0.48,1.02	0.91,1.23	0.87,1.56	0.93,1.56	0.48,1.04	0.40,1.68	0.38,5.15	0.17,22.52	
	0.594	0.362	0.172	0.064	0.441	0.315	0.156	0.080	0.594	0.610	0.601	
	0.599	0.428	0.263	0.126	0.499	0.39	0.247	0.151	0.599	0.607	0.603	
Side road density	0.92	0.91	0.90	1.05	0.91	0.88	0.90	1.03	1.14	1.46	0.90	–
	0.84,1.02	0.75,1.09	0.75,1.08	0.84,1.32	0.83,1.01	0.72,1.07	0.75,1.09	0.82,1.31	0.76,1.69	0.76,2.83	0.42,1.92	
	0.098	0.306	0.257	0.669	0.072	0.188	0.298	0.776	0.525	0.259	0.782	
	0.172	0.384	0.34	0.621	0.139	0.281	0.378	0.689	0.541	0.341	0.69	
Residence density	0.61	1.87	0.91	0.34	0.58	1.70	0.67	0.34	10.84	7.57	57.09	–
	0.19,1.88	0.34,10.48	0.12,7.20	0.01,8.70	0.18,1.81	0.30,9.75	0.08,5.50	0.01,9.98	0.01,11884.4	0,47684.59	0,4361582	
	0.395	0.474	0.932	0.515	0.346	0.551	0.706	0.530	0.505	0.650	0.481	
	0.454	0.512	0.763	0.541	0.417	0.560	0.645	0.541	0.533	0.618	0.518	
Public park density	0.58^***^	0.88	0.67^*^	0.42^***^	0.56^***^	0.81	0.68	0.37^***^	1.86	3.49^**^	3.49	–
	0.49,0.69	0.65,1.19	0.48,0.92	0.26,0.69	0.46,0.66	0.59,1.11	0.49,0.96	0.22,0.62	0.77,4.51	1.49,8.14	0.02,617.36	
	0.000	0.412	0.014	0.001	0.000	0.194	0.026	0.000	0.167	0.004	0.636	
	0.001	0.472	0.039	0.004	0.001	0.285	0.062	0.001	0.26	0.014	0.609	
Distance to school	1.11	1.20	1.46	0.85	1.15	1.41	1.26	0.96	0.85	0.68	2.44	–
	0.92,1.34	0.87,1.66	1.02,2.08	0.46,1.56	0.93,1.41	1,1.99	0.85,1.87	0.47,1.96	0.46,1.55	0.17,2.68	0.1,61.11	
	0.285	0.261	0.037	0.597	0.191	0.047	0.248	0.905	0.602	0.581	0.588	
	0.363	0.341	0.082	0.602	0.285	0.098	0.334	0.74	0.603	0.598	0.598	
Constant	50.19	78.56	313.79	11.95	56.32	67.65	421.71	15.39	0.10	29.12	0.01	-

a *All models were adjusted for age, gender, neighborhood socioeconomic status, and urbanicity*.

b *The overall model reports the result of a logistic mixed regression model including a random effect for the three waves. The Wave 1–3 models reports the results of logistic regression models for wave 1–3, respectively. Wave 1 represent the cohort that took the health survey from 2011 to 2012; Wave 2 represent the cohort that took the health survey from 2013 to 2014; Wave 3 represent the cohort that took the health survey from 2015 to 2016*.

c *For each variable, the first row reports the odds ratio; the second row reports the 95% confidence interval, the third row reports the naïve p-value; the fourth row reports the FDR sharpened q-value*.

d *p < 0.05 and q < 0.02, **p < 0.01 and q < 0.03, ***p < 0.001 and q < 0.004.

After FDR adjustment, children living in neighborhoods with high density of Chinese fast food restaurants showed a lower BMI (Table 2) and were less likely to be overweight (Table 3) and obese (Table 4), while those with high density of Western fast food restaurants showed a higher BMI (Table 2) and were more likely to be overweight (Table 3) and obese (Table 4). The associations of high density of Chinese fast food restaurants and low BMI and overweight/obesity risk were significantly observed in 2011–2012 (Tables 2–4) and 2013–2014 (Tables 2–4) but not significant in 2015–2016. In contrast, the associations of high density of Western fast food restaurants and high BMI and overweight/obesity risk were not observed significant until 2015–2016 (Tables 2–4).

### Built Environment

After FDR adjustment, children living in neighborhoods with high land use mix level were less likely to be overweight (Table 3) but the association was only observed significant in 2011–2012 (Table 3). After FDR adjustment, children living in neighborhoods with high density of main roads showed higher BMI (Table 2) and were more likely to be overweight (Table 3) but the association was only observed significant in 2011–2012 (Tables 2, 3), while children living in neighborhoods with high density of side roads showed lower BMI (Table 2) and were less likely to be overweight (Table 3) but the association was only observed significant in 2011–2012 and 2013–2014 (Tables 2, 3). After FDR adjustment, children living in neighborhoods with high park density showed lower BMI (Table 2) and were less likely to be overweight (Table 3) and obese (Table 4). The associations were observed consistent in all three time periods and were observed significant in 2013–2014 (Tables 2–4) and 2015–2016 (Tables 2–4). After FDR adjustment, children living in neighborhoods with longer distance to school were less likely to be overweight (Table 3) but the association was not observed for BMI nor obesity risk. No significant associations were observed between residential density and childhood body size.

### Associations of Neighborhood Environment and Weight Status Among Primary and Middle School Children

The right parts of Tables 2–4 showed the associations of neighborhood built environments and child body size among primary and middle school children, respectively. (1) After FDR adjustment, both primary and middle school children living in neighborhoods with higher SES tended to have lower BMI (Table 2), while the associations were not observed significant between neighborhood SES and overweight/obesity (Tables 3, 4). (2) No significant associations of urbanity and childhood weight status were observed among primary nor middle school children.

### Food Environment

After FDR adjustment, primary school children living in neighborhoods with high density of fruit/vegetable markets showed a lower BMI (Table 2) and less likely to be overweight (Table 3), while the association was not observed significant for obesity risk. The associations of high density of fruit/vegetable market and low BMI and low overweight risk among primary school children were significantly observed in 2011–2012 (Tables 2, 3) and 2013–2014 (Tables 2, 3) but not observed significant in 2015–2016. The associations were not observed significant among middle school children. After FDR adjustment, the associations of supermarket density and childhood weight status were inconsistent between years. In 2011–2012, both primary (Tables 2, 3) and middle school children (Tables 2, 3) living in neighborhoods with high supermarket density showed a higher BMI and were more likely to be overweight. In 2013–2014, primary school children living in neighborhoods with high supermarket density showed a lower BMI (Table 2) and were less likely to be overweight (Table 3), while the associations were not observed significant among middle school children in this wave.

After FDR adjustment, primary school children living in neighborhoods with high density of Chinese fast food restaurants showed a lower BMI (Table 2) and were less likely to be overweight (Table 3), and obese (Table 4), while those with high density of Western fast food restaurants showed a higher BMI (Table 2) and were more likely to be overweight (Table 3) and obese (Table 4). Middle school children living in neighborhoods with high density of Chinese fast food restaurants were less likely to be overweight (Table 3), while the significant associations were not observed for BMI nor obesity rate. No associations were observed significant between the density of Western fast food restaurants in the neighborhood and middle school children body size. The associations of high density of Chinese fast food restaurants and low BMI and overweight/obesity risk were significantly observed among primary school children in 2011–2012 (Tables 2–4) and 2013–2014 (Tables 2–4), but disappeared among primary school children in 2015–2016. In contrast, the associations of high density of Western fast food restaurants and high BMI and overweight risk were not observed significant among primary school children in 2011–2012 or 2013–2014 but were observed significant in 2015–2016 (Tables 2, 3), while the associations of high density of Western fast food restaurants and high BMI and overweight/obesity risk were not observed among middle school children. No significant associations were observed between convenient store density and childhood body size among primary nor middle school children.

### Built Environment

After FDR adjustment, no significant associations were observed between high land use mix level and childhood body size among primary nor middle school children. After FDR adjustment, no significant associations were observed between high density of main roads and childhood body size among primary nor middle school children, while primary children living in neighborhoods with high density of side road showed lower BMI (Table 2) and were less likely to be overweight (Table 3) but the association was only observed significant in 2011–2012 (Tables 2, 3). The association was not observed among middle school children. After FDR adjustment, both primary and middle school children living in neighborhoods with high park density showed lower BMI (Table 2) and were less likely to be overweight (Table 3) or obese (Table 4). Middle school children living in neighborhoods with long distance to school were less likely to be overweight but the association was only observed in 2015–2016 (Table 3). The association was not observed among primary school children.

## Discussion

This is a retrospective time-trend study using healthy survey data of a mid-sized city in China to investigate the relationship between childhood obesity and different factors of the neighborhood built environment, including fast food restaurants, fruit/vegetable markets, public parks, and road density. It is among the first quantitative studies separately discussing the impacts of Western-style and Chinese-style fast food environments upon childhood body size. The results show that Western-style and Chinese-style fast food have divergent impacts on childhood body size. Paralleling with studies on Western countries (18, 26, 32) increased exposures to Western-style fast food may increase child BMI and the risk of overweight and obesity. In contrast, increased exposures to Chinese-style fast food may reduce child BMI and the risk of overweight and obesity, indicating a positive health impact of Chinese fast food.

However, urban neighborhood environment may have gradually reduced the positive health impact of Chinese-style fast food, as the negative association between Chinese-style fast food and childhood body size were only observed in 2011–2014 and were gradually disappeared in 2015–2016. Instead, the positive association between Western-style fast food and childhood body size became significant while the impact of Chinese-style fast food declined. In other words, the positive health impact of Chinese-style fast food has been gradually replaced by the negative health impact of Western-style fast food. The trend is in parallel with the strong presence of Western-style fast food in urban China's food catering market (36) and the popularity of Western-style fast food has dramatically reshaped the ways how Chinese-style fast food is cooked (37). Most importantly, the fast-paced lifestyle resulting from rapid urbanization is gradually transforming Chinese urban residents' food consumption patterns, from regularly scheduled, home-made Chinese meals to frequent consumption of fast food, shopping from modern supply chains and snacking multiple times a day. Fast food that features speediness, high energy and convenience is ideal for such lifestyle. More quantitative analyses are needed to provide more concrete categorization on Chinese-style and Western-style fast food in urban China and to further investigate the healthiness of particular types of fast foods, the appropriate amount to consume for children and adults per meal to maintain a balanced energy/nutrition intake, as well as their specific health impact on body size and risks of certain diseases.

The positive health impact of fruit/vegetable markets has also been reduced in the urban neighborhood environment. Paralleling with studies in the U.S. (26), increased exposures to fruit/vegetable markets may reduce child BMI and the risk of overweight and obesity, although such effects were only observed in 2011–2012 and were gradually disappeared in 2013–2016 along with the healthy effect of Chinese fast food. With the rapid real estate development in Zhanjiang since 2008, traditional neighborhoods were generally replaced by modern residences and businesses. Meanwhile, it has become more difficult for traditional fruit/vegetable markets to afford the ever-rising land rent. In addition, the rise of fast food delivery services in recent years has further threatened the existence of fruit/vegetable markets. The cheap capital dominated the produce supply chain and lower quality of food in the fruit/vegetable market (38). To improve children's accessibility to fresh and healthy food, planning and policy strategies should be made to encourage sustainable development of healthy food supply and to reform traditional fruit/vegetable markets for modernized China. Future studies should also estimate the impact of the rapid development of convenient stores and fast food delivery services on people's health, qualitatively reveal how such development affects the diet, energy/nutrition intake, and the risk of related diseases of Chinese people, and also to quantitatively evaluate the extent to which it changes the quantity of energy/nutrition intake and the prevalence of related diseases in contemporary China.

This study is also among the first to simultaneously consider the impact of food and physical activity environment upon childhood body size in China. The density of main roads and side roads indicates the walkability of a neighborhood. Children living in neighborhoods with higher density of main roads and lower density of side roads, which imply lower walkability, were demonstrated to be obesogenic in 2011–2014. However, the associations disappeared in 2015–2016, which may be related not only to the rapid urban development, but also to the dominance of cheap capital which brought low-quality and high-risk food supply chain (37). Cheap food stores of such kind more often spread along the side roads in urban China (41, 42), which may obscure the positive effects of side road density in promoting physical activities and low body size. This study also parallels with studies in Western countries (13, 14, 25) and demonstrates the positive effect of public park exposure and distance to school in promoting physical activity so as to reduce the risk of overweight and obesity. The findings suggest to urban planners the importance of providing neighborhood accessible public parks, proper school density and other types of physical activity facilities when conducting “small-block size and dense road-network” project, and also the importance of regulating the development of inexpensive food stores along the side roads.

Last but not least, this study reveals the divergent influences of neighborhood built environment around residence between primary and middle school children. The findings demonstrate that food environment around residence have more influence on the body size of primary school children while the influence was not largely observed among middle school counterparts. This may be due to the fact that most of the middle schools in China provide lunches and/or dinners to students. Middle school children's daily food intake and physical activity may be much affected by the neighborhood built environment around school. In addition, longer distance to school may only reduce the overweight risk of middle school children but the influence was not largely observed among primary school counterparts. This may be due to the fact that a large proportion of middle school children walking or cycling independently to and from schools every day. For primary school children, their school commutes are usually accompanied by parents, with a large number of them driving or riding their children by private vehicles or bicycles. Hence, commuting to school does not increase primary school children's physical activities. This finding suggests that more attention should be paid to school and its surrounding built environment when examining the environmental causes of middle school children obesity. For primary school children, neighborhood food environment and active school commute are key factors to prevent childhood obesity. Future studies should examine the barriers to primary and middle school children's active school commutes so as to lower or remove such barriers in future urban planning.

There are some limitations of this study. First, this is a retrospective time-trend study on a mid-sized city in Guangdong Province, China. Although comparisons have been made between the findings of this study and other studies in China and worldwide, more case studies should be conducted in different locations, in cities of different sizes, and in rural areas to provide more evidence for the patterns and impacts of neighborhood built environment on childhood body size. Although this study uses a health survey data of 6 years, only the built environment data in 2016 is available, which cannot represent the change of the built environment from 2010 to 2016. Furthermore, the definition of childhood overweight and obesity is based on the national growth chart WS/T586-2018. However, the prevalence of overweight and obesity among primary school children suggested that the mean age of puberty onset of the observations in our study may be younger than the national average. Future studies should pay more attention to the diverse growth chart among children in different parts of China and provide accurate definition of childhood overweight/obesity. In addition, some sociodemographic risk factors, such as household income, and risk behaviors, such as diet and physical activity, are not provided in this dataset (6), which might be possible explanation for the residual association observed.

## Conclusion

In conclusion, this study demonstrates the significant impacts of walkability, as well as the neighborhood food environment, which does not only include the spatial accessibility of food but also the quality of a healthy food supply chain upon childhood body size in a mid-sized city of China. It builds on the built environment and childhood obesity literature by examining the divergent impact of Chinese-style and Western-style fast food exposure, and revealing the divergent impact of neighborhood built environment between primary and middle school children. The findings from this study provide informed implications for neighborhood level urban planning and design in promoting the health of children and adolescents. Besides the widely recognized features of healthy urban form such as walkable neighborhood with accessible green/blue spaces, fruit/vegetable markets and Chinese-style fast food show the potential to be important characteristics of healthy food environment. However, such potential is threatened by the fast-paced lifestyle, competition from Western-style fast food, and low-quality, high-risk food supply chain coming along with rapid urbanization. Municipal food and drug administrations should regulate the rapid development of fast food restaurants, both Chinese and Western styles, in urban China. Future urban planning and design should pay more attention to the food environment as well as physical activity environment, especially on neighborhood healthy food environment, which are proved to be essential for children health. More attention should also be paid to food and physical activity environment around school as they are also essential for children health, middle school children in particular. A comprehensive healthy food environment, including not only a food accessible neighborhood but also a healthy food supply chain, along with walkable built environment are essential to counteract the health risks associated with rapid urbanization.

## Data Availability Statement

The datasets generated for this article are not readily available because The datasets generated and/or analyzed during the current study are not publicly available due to the contract between the researchers and data provider but are available from the corresponding author on reasonable request. Requests to access the datasets should be directed to Kun Liu, liuk@hit.edu.cn.

## Ethics Statement

This study was approved by the ethics committee of Zhanjiang Disease Prevention and Control Center and Harbin Institute of Technology (Shenzhen) Internal Review Board, which are in accordance with the Declaration of Helsinki. All participants provided informed consent according to these procedures prior to any data collection. Informed consent was provided from a parent and/or legal guardian of the participants under 18.

## Author Contributions

PZ drafted the manuscript and undertook the statistical analysis. PZ, RL, and KL developed the whole research designs, contributed to the interpretation of the results, and reviewed the manuscript. KL undertook the data collection. All authors read, revised, and approved the final manuscript.

## Conflict of Interest

The authors declare that the research was conducted in the absence of any commercial or financial relationships that could be construed as a potential conflict of interest.

## Publisher's Note

All claims expressed in this article are solely those of the authors and do not necessarily represent those of their affiliated organizations, or those of the publisher, the editors and the reviewers. Any product that may be evaluated in this article, or claim that may be made by its manufacturer, is not guaranteed or endorsed by the publisher.
